# Heteronormative biases and distinctive experiences with prostate cancer among men who have sex with men: a qualitative focus group study

**DOI:** 10.1186/s12894-023-01398-0

**Published:** 2024-01-06

**Authors:** Evan Panken, Noah Frydenlund, Neil Mistry, Rahul Prabhu, Jeffrey Wong, Shilajit Kundu, David Victorson, Channa Amarasekera

**Affiliations:** 1 Department of Urology, Northwestern University, 675 N. St. Clair St, Chicago, IL 60611 USA; 2 Department of Medical Social Sciences, Northwestern University, Chicago, IL USA; 3 University of Colorado School of Medicine, Aurora, CO USA

**Keywords:** Men who have sex with men, Qualitative, Focus groups, Prostate cancer, Sexual dysfunction, Ejaculatory dysfunction, Prostate cancer survivorship, Communication with health professionals

## Abstract

**Background:**

Men who have sex with men (MSM) face many challenges and biases in healthcare. Within urology there is a need to better understand how prostate cancer impacts MSM given the unique ways in which side effects that accompany treatment may affect this population. The goal of this study is to explore the experience of MSM with prostate cancer to advance the existing literature in this area and inform implementation and delivery of clinical practice and policy guidelines.

**Methods:**

Four focus groups were conducted with a semi-structured interview guide. Using a phenomenological qualitative approach consistent with grounded theory [1] and naturalistic inquiry principles we sought to better understand the direct experiences of MSM with prostate cancer. Audio transcriptions were thematically analyzed to identify themes that impact MSM throughout their prostate cancer journey. An iterative, team-wide classification process was used to identify, organize, and group common codes into higher-order categories and themes.

**Results:**

Patient’s choice of provider and their interactions with the healthcare system were strongly impacted by their sexual identities. Participants commented on navigating the heteronormative healthcare environment and the impact of assumptions they encountered. MSM experienced the sexual side effects of prostate cancer treatment in unique ways. Issues with erectile dysfunction and ejaculatory dysfunction had significant impacts on patient’s sexual experience, with some describing being forced to explore new modes of sexual expression. Anejaculation was a theme that was distressing for many participants. The emotional impact of a prostate cancer diagnosis was significant in the men interviewed. Common themes included loss of identity and fear for future relationships.

**Conclusions:**

MSM have unique concerns after prostate cancer treatment that differ from men who don’t identify as MSM. It is critical that providers familiarize themselves with the concerns of this patient population regarding prostate cancer treatment. An important step toward reducing heteronormative bias in prostate cancer care is to better understand the goals, identity, and sexual practices of MSM and to provide informed anticipatory guidance.

**Supplementary Information:**

The online version contains supplementary material available at 10.1186/s12894-023-01398-0.

## Background

The heteronormative nature of society leads to daily challenges for the LGBTQ + population [2–5]. This bias extends to medical care, with LBGTQ + patient populations facing a multitude of barriers to services [6]. Many have difficulty accessing care, experience prejudice in health care encounters, and are at higher risk for worse health outcomes compared to their heterosexual peers [3, 7, 8]. There has been an increase in calls for research into the barriers, experiences, and outcomes for this patient population across medical specialties [9–11]. Within urology and the prostate cancer space in particular, there has been increasing research into the experiences of men who identify as gay, bisexual, or as men who have sex with men (MSM), given the unique ways in which side effects that accompany treatment may affect these populations [12, 13].

MSM serves as a useful epidemiological category as it includes both men identifying as gay or bisexual (GBM) and men who do not identify as gay or bisexual but engage in sexual relations with other men. In urology, this category allows research to capture both identity-based bias faced by GBM patients as well as behavior-based bias faced by MSM patients. While the former may become apparent in physician attitudes and structural discrimination, the latter refers to heterosexual bias when it comes to diagnosis, counseling, and treatment that may impact physician decision-making [14, 15].

It is estimated that between 50,000 and 198,000 MSM are currently living with prostate cancer in the United States (US) [16]. Conflicting evidence has shown both lower and higher rates of prostate cancer in MSM as compared to men who don’t identify as MSM, but these studies suffer from low sample-sizes [17–19]. It is important to consider that there was a large loss of life of sexual and gender minority individuals from the HIV/AIDS epidemic in the 1980 and 1990 s, and as this has become a treatable condition there will be more MSM reaching the ages when prostate cancer is most commonly diagnosed [20]. Additionally, men in same sex relationships have double the risk of experiencing prostate cancer as a couple, and for this reason it is important to study how prostate cancer is experienced and managed by these men. Overall, although the amount of research into MSM with prostate cancer has increased, there is a disproportionate amount of research into MSM patients’ experiences with prostate cancer and its treatments, and how these might differ from men who don’t identify as MSM. Two survey-based studies found that many MSM feel that sexual function is assessed incompletely with current validated questionnaires, raising the importance of understanding the unique concerns of this population [21, 22]. The concept analysis by Mitchell et al. identified 4 essential elements of sexual dysfunction among prostate cancer survivors who identified as GBM and postulated that sexual dysfunction is experienced distinctly by this patient population [23]. A better understanding of the experiences of MSM with prostate cancer will allow providers to create a more accessible and inclusive clinical environment. This in turn can lead to more effective shared decision making and opportunities to better counsel MSM on available treatment options by addressing cultural and population specific needs and concerns.

There have been calls to diversify the methods for researching LGBTQ + health, to move beyond a deficits-based approach and involve the LGBTQ + community’s voices in describing their own lived experiences [24]. Qualitative research is a form of rigorous scientific inquiry which allows investigators to hear directly from patients and ensure that patient experiences are accounted for and incorporated. A few qualitative studies have begun to elucidate experiences of gay men with prostate cancer, finding that they may have unique considerations that should be addressed including worry about disclosing their sexuality, a deficit in culturally competent resources, and a lack of appropriate counseling on side effects in the context of their identity [13, 25–28]. One study of 11 gay men in Sweden who had been treated for prostate cancer identified themes including the importance of ejaculate, changes in their body affecting relationships, and they emphasized the importance of focusing on sexual practice, having sex with men, rather than sexual identity, gay or bisexual, when considering rehabilitation programs and appropriate counseling [29]. This study aims to add to the available qualitative data on the experiences of sexual minorities with prostate cancer through the use of a phenomenological qualitative approach. This study captures a wider range of themes owing to its more inclusive focus on MSM, focusing on sexual behavior and including different gender and sexual identities. MSM is a group that is defined by sexual activity and includes a wide range of gender identities and sexual orientations.

The purpose of this current qualitative study is to explore the experience of MSM with prostate cancer to advance the existing research literature in this area and inform implementation and delivery of clinical practice and policy guidelines.

## Methods

### Participants and eligibility

This study was approved by the governing Internal Review Board at a large, Midwestern University. Participants were eligible if they met the following inclusion criteria: (a) self-reported as MSM; (b) diagnosed with prostate cancer; (c) over the age of 18; (d) able to read and communicate in English and understand and provide informed consent. Our general exclusion criteria included: (a) men under the age of 18 and over the age of 89; (b) severe cognitive impairment as determined by the referring medical team member leading to an inability to understand and provide informed consent.

### Setting and recruitment

We identified and enrolled eligible participants by mailing invitations to urology clinics in Chicago, posting flyers in LGBTQ + establishments such as bars and health clinics, and mailing lists for LGBTQ + social organizations. Potential participants underwent screening by phone to ensure they met the inclusion criteria. We sought to include a heterogeneous MSM sample to ensure diversity of perspectives and experiences that included representation in terms of demographic and clinical characteristics, time since diagnosis, prostate cancer treatment modalities, sexual and urinary symptoms, relationship status, income, and education.

### Procedures

We conducted four audio-recorded, 90-minute in-person focus groups. Focus groups are an appropriate qualitative data collection method to quickly and efficiently gather information from purposefully selected groups of people, whose reflections and responses spur additional discussion around facilitated topics [30]. The number of focus groups in our study was selectively chosen in order to achieve thematic saturation based on the recommendations by Guest et al. [31]. Prior to starting the group, participants were asked to complete a survey that assessed socio-demographic, clinical and sexual health characteristics to describe the group. Participants were assigned different color cards to maintain anonymity and to assist with analysis. Focus groups were moderated by experienced facilitators (CA, DV). A semi-structured moderator’s guide was developed to help direct discussion and can be viewed in the additional file (Additional file 1). Discussion began following a brief orientation to the study and review of ground rules. The following probes were available to moderators to guide discussions if needed: 1) “Tell us how your life has been affected by prostate cancer?”; 2) “Prostate cancer and its treatment can produce different sexual symptoms and side effects. What kinds, if any, were the most common for you?”; 3) “How did you (or would you) deal with or manage with changes in sexual function?”; 4) Please take a moment to review these questionnaires that currently assess sexual function in men after treatment for prostate cancer.”; 5) “Is there anything else that you think is important for me to know about your experience that I did not ask you about?”. Questions were open ended and elicited responses related to participants’ experiences with prostate cancer. We reviewed the quality of all audio recordings prior to verbatim transcription (excluding identifying information). The research coordinator contacted participants who completed the study to thank them for their participation and informed them of their right to receive information on de-identified group-level findings upon study termination. Participants were compensated twenty-five dollars each and parking was reimbursed.

### Analysis

Verbatim audio transcriptions were analyzed using the software Dedoose©. Using a phenomenological qualitative approach [32] consistent with grounded theory [1] and naturalistic inquiry principles [33] we sought to better understand the direct experiences of MSM with prostate cancer. We were specifically interested in learning about their interactions with the healthcare system and lasting effects of treatment. To ensure the rigor of our findings we utilized several qualitative research strategies to enhance credibility and dependability, including (1) Review of transcript accuracy against recordings; (2) Creation of a code book based on an initial phase of open coding by investigators (EP, NF, NM); (3) Assuring adequate inter-rater reliability of coding across raters prior to coding (kappa ≥ 0.70 was used as an acceptable level of agreement); (4) Engaging in an axial and selective coding with remaining transcripts, creating new codes as they emerged; (5) Engaging in an iterative, team-wide classification process to identify, organize, and group common codes into higher-order categories and themes.

## Results

In total, 26 men participated across four focus groups (Group I = 6; Group II = 7; Group III = 6, Group IV = 7). All participants identified as MSM and had a diagnosis of prostate cancer. The median age was 70.7 years old (range = 55–84). The cohort was predominantly white (n = 25) and born in the United States (US) (n = 25). The highest level of education achieved ranged from high school graduate/GED (n = 2), one or more years of college without a degree (n = 4), associate’s degree (n = 2), bachelor’s degree (n = 10), master’s degree (n = 6), to doctorate degree (n = 2). Additional demographic information can be found in Table 1.

**Table 1:** Participant Demographics

**Age (years)**	Participants (%)
55–60 61–65 66–70 71–75 ≥76	8% 12% 28% 24% 24%
**Race (n = 26)**	
White	96%
**Education**	
High school graduate/GED One or more year of college, no degree Associates degree Bachelor’s degree Master’s degree Doctorate degree	8% 15% 8% 38% 23% 12%
**Marital Status**	
Single Married Living with partner Widowed Divorced	50% 31% 8% 4% 8%
**Income in Previous Year**	
5-9.9 K 10-19.9 K 20-39.9 K 40-74.9 K 75-99.9 K 100 K or more	4% 0% 15% 38% 19% 23%

In total we applied 187 unique codes to the transcripts, using exhaustive coding, 1346 times across the four focus groups. The inter-rater reliability kappa statistic was > 0.7 for all coders. Saturation was achieved at the theme level (Additional file 2). Ultimately, six overarching themes were identified through our analysis: (1) Initial diagnosis and treatment planning, (2) Identity and preferences, (3) Positive and negative experiences with care, (4) Communication, (5) Sex and intimacy, and (6) Life perspective after treatment. Each theme is described below with exemplar participant quotations to illustrate its significance.

### Theme 1: initial diagnosis and treatment planning

This theme focused on patients’ reactions to their initial diagnosis of prostate cancer and how they remember discussions of prognosis and treatment. This theme was characterized by the categories “feelings towards cancer diagnosis”, “patient recount of prognosis discussion”, “provider recommendations”, and “social factors impacting treatment decisions”. There were a wide range of feelings that participants experienced with their initial cancer diagnosis. Some participants noted a feeling of hopelessness with one person saying,

*“You feel like your life is over, and you feel like this is really it, and there’s nothing to live for anymore. I mean, I went through these things very quickly” (Pink, FG2)*.

Other participants felt fear or anger when they received their initial diagnosis, and many noted a sense of shock. One patient discussed that the shock of receiving the diagnosis of prostate cancer led him to face his own mortality noting,

*I was diagnosed 2 years ago, and it kind of shattered my myth of almost immortality. Here I was, 73, the only medicine I took was baby Aspirin, I would go to the gym, bike and whatever, and then all of the sudden got the diagnosis” (Orange, FG1)*.

Some participants remember accepting the diagnosis of cancer and being motivated to take action,

*“When I was told that it was cancer, I just said “Okay, what do we do next?” I’d love to say it affected me a little bit more than it did, but I said, “Okay, I got it.” Let’s go onto the next step to cure it.” (Green, FG2)*.

### Theme 2: identity and preferences

This theme focused on participants’ goals and treatment preferences as well as how their sexual identity informed these decisions. This theme was characterized by the categories “alternative medicine”, “patient goals of treatment”, “patient treatment preferences”, “preferences about healthcare providers”, and “sexual identity”. Sexual identity played a key role in healthcare interactions and many participants preferred a gay primary care physician (PCP), because they felt that a gay physician would relate to them better. One participant spoke about the shared understanding a gay physician has,

*“for a GP (general practitioner) I like having a gay male doctor and I sought out a gay male doctor because I just feel the state of mind, they know our needs, they understand it, you don’t have to explain it, and he asks me questions that I don’t think a heterosexual doctor, male or female would know to ask. He knows; he understands, period.” (Pink, FG2)*.

Another participant spoke about the importance of seeing a gay physician saying,

*“There are many, many great doctors out there, but for me being a gay man, it was important for me to have a gay doctor, and I have a wonderful doctor who is gay.” (Green, FG2)*.

Although many participants had a strong preference for a gay PCP, this didn’t seem to be as consistent when it came to urologists. Many participants said that the sexual orientation of their urologist wasn’t the important part, rather participants were more focused on the technical competency of their urologist and feeling supported and understood during visits. One participant noted,

*“I just want to say I think there’s a commonality we are hearing; I’m okay with my Urologist, I don’t know what my Urologist’s sexual preference is, all I knew is that he was a great surgeon, I loved his demeanor, I loved how he gave me an hour consultation, my family was with me. I felt very in good hands with him. But, for a GP, I have a gay male doctor and it makes all the difference; I feel much more comfortable.” (Pink, FG2)*.

### Theme 3: positive and negative experiences with care

This theme encompasses the participants’ interactions with the healthcare system for the management of prostate cancer as well as their feelings towards their post-treatment sexual function. It was characterized by the categories “dissatisfied with medical care”, “lack of side effects after treatment”, “negative feelings towards health care providers”, “negative interaction with health care provider”, “positive feelings towards health care providers”, “preserved sexual function after treatment”, “satisfied with medical care”, and “satisfied with sexual function after treatment”. One participant discussing the importance of the physician-patient relationship said,

*“I have to say for me, I didn’t feel any kind of bias or prejudice, it’s getting the right doctor that asks the right questions to really get to know you. I could see how it could be very unpleasant if they don’t, so make it personal; understands your lifestyle, understands your needs, can talk to you openly.” (Pink, FG2)*.

Participants spoke about their experiences with the heteronormative environment of clinics and the assumptions they encounter,

*“I just wanted to say, you’re treated like you’re heterosexual… everyone just assumes you’re heterosexual, period… unless you say something, they’re just assuming you’re married to a woman, you’re sleeping with a woman, and you’re heterosexual, end of story” (Pink, FG2)*.

Patients found this environment challenging and uncomfortable, one participant discussed the reaction of his urologist when disclosing his sexual orientation,

*“I just never felt comfortable. And, in a discussion with the Urologist himself, when I mentioned I was gay, he acted surprised. He said, “Well I don’t care.” But that just seems kind of odd.” (Grey, FG2)*.

Many participants discussed having a positive relationship with their medical providers. Other participants discussed difficult and frustrating encounters with their care. Some of the sources of frustration included a sense of not being listened to, lack of sufficient information, and poor communication, with one person saying,

*“I think that was one of the major failings I thought along the office structure of my medical people. I mean, nowhere did they have any counselling; this is what’s going to be what happens to your partner, this is your job, or not your job, but you’re not going to break him. He’s already on the mend, and I think there’s a lot of—not even misunderstanding, it’s just no communication whatsoever on anyone’s part” (Pink, FG1)*.

Other participants spoke about how a positive relationship or interaction during their medical care was greatly therapeutic. One person said,

*“The trust level was there from the minute I walked in. Staff; incredible. His assistant, everyone just made you feel so comfortable. And, the honesty factor was there, the trust, the honesty. He went through things, different procedures for me, didn’t push anything on me, but the way he explained the different procedures turned totally from wanting to have the radiation or the beads or whatever to, “Let’s just take it out,” in fifteen minutes I made the decision, let’s just go for it.” (Green, FG2)*.

### Theme 4: communication

This theme concerns the many places throughout the treatment process where the participants highlighted the importance of communication and support. This theme consisted of the categories “disclosure of sexual orientation”, “discussing prostate cancer with family”, “education about prostate cancer”, “negative communication with partner”, “patient support systems”, “positive communication with partner”, and “ways in which partners are supportive”. It includes positive and negative communication experiences with partners, disclosure of their prostate cancer, support systems they leaned on, and the many ways they received or gave education on prostate cancer. Relationships as a strong source of support was a consistent topic, with one participant saying,

*“Based on how he responded, and how he cared for me through all that, how he stayed with me, gave me the sign; this is someone that I should probably spend my whole life with. At that point, it’s a special person that could stay with someone through prostate cancer and all the side effects that come afterwards, which is significant. So, I would say it’s a test of the character, and if you’re dating someone and they can’t take it, that wasn’t someone that was for you in the first place.” (Yellow, FG2)*.

Other participants who were not in a supportive relationship discussed how they wished for a relationship or thought that it would make the prostate cancer process easier, with one saying,

*“I think perhaps going through all this trauma is easier for a partnered person to go through than it is for somebody that is single. I really believe that…[a] support person that is showing that it isn’t going to affect your security with him, where as a single person is wanting to have… a partnership and it’s not there right now, and I think that what we are seeing here is that it’s easier for a partnered person to go through this prostate process than it is for a single person, and I understand and appreciate that. (Red, FG1)*

Many participants pointed out choosing to disclose their sexual identity to their support systems or physicians as an important theme in their experiences with prostate cancer, one saying,

*“Oh, that’s the first thing that comes out of my mouth to any doctor that I see, I tell them, “And by the way, I am a gay male.” And they say, “Oh, what difference does it make?” And I said, “I’m not sure, but it might; depending on what areas we have to deal with, but I tell them right up front whether they want to know or not.” (Green, FG1)*.

One sentiment that recurred throughout the focus groups was the idea of honesty with one participant saying,

*“I don’t know how the rest of you feel, but in today’s world, the honesty thing has become much more important to me than it ever was before, as I see our world sort of devolve away from honesty as a basic principle of how we function.” (Blue, FG3)*.

### Theme 5: sex and intimacy

This theme focuses on the way the participants’ treatment impacted their sexual function and sexual experiences. It was composed of the categories “changes in sexual experience after treatment”, “change in sexual practices after treatment”, “concerns surrounding sex”, “distinguishing intimacy and sex”, “erectile issues”, “negative experiences with incontinence”, “negative partner reaction to sexual dysfunction”, “partner less interested in having sex”, and “side effects from radiation.” While patients faced a significant loss in pre-treatment sexual function and felt this loss deeply, many adapted and found alternative means of positive sexual experiences. The large proportion of single men within our participants and among MSM in general made navigating these changes particularly difficult. One participant said,

*“The word that came to mind was damaged; I called and said, “I’m damaged goods.” I’m malfunctioning. It’s hard enough when you’re healthy to get someone decent and date. I kind of stopped because I got sick of everything and did other things, but I felt like the curtain closed on that stage, that’s just over with.” (Pink, FG2)*.

For some participants this change was felt as inadequacy and had an impact on their sense of masculinity. One participant reflected,

*“I identify with a lot of what you said in terms of feeling inadequate at this point. Feeling less of a man at this point. That’s frustrating for me.” (Green, FG2)*.

Changes in sexual function ranged from loss in erectile function and urinary incontinence to depleted ejaculatory function. Effects on orgasm stood out as a prominent obstacle with one participant saying,

*“Dry orgasm is a conversation point, if we get to the point where we’re going to have sex, because I’m thinking, I feel I need to prepare somebody that you’re not going to get a surprise at the end.” (Blue, FG1)*.

One participant commented on how this was a result of cultural aspects surrounding his sexuality as a gay male, saying,

*“I think we all came into our situations with our experiences from adolescence and on where it was furtively jacking off to what you could, and the whole focus of our sexual lives growing up was the ejaculation.” (Red, FG2)*.

Participants reflected on having to not only process this loss but also adapt to these changes to find sexual pleasure through alternative means. For some, this involved making a switch from the insertive role (top) to a receptive role (bottom) or oral sex. One participant said:

*“There have been, due to necessity, modifications to be made when I’m intimate with someone. I’m very oral. I always have been in that regard, but to be honest with you I was never a bottom.” (Green, FG2)*.

For other participants the changes they experienced forced them to discover new avenues for sexual satisfaction that they welcomed as an opportunity. One participant describes this as,

*“There are things that we do now, areas that we concentrate on the body that I never concentrated on before that turn out to be equally if not more satisfying…. And, I have wondered if we would have, if I would have gotten there as easily without the prostate cancer… prostate cancer kind of opens up to looking around our body a little better.” (Red, FG3)*.

### Theme 6: life perspective after treatment

This theme encompasses how our participants carried their experiences with prostate cancer with them as they got further removed from the initial diagnosis and treatment. This theme was characterized by the categories “acceptance of side effects”, “anxiety about future relationships”, “fear of recurrence”, “optimism for prostate cancer and the future”, “recovering from side effects”, and “understanding implications of age”.

This included improvement and acceptance of side effects, as well as fear, anxiety, and optimism for the future. Many participants expressed a sentiment of acceptance and learning to live with their treatment side effects, one example being,

*“And I just thought… what could you do? There was a lot of stuff you could still do, so you kind of have to let yourself evolve, you have to get creative with it, and so I no longer think of myself as damaged anymore,” (Pink, FG2)*.

Other participants discussed anxiety and concerns about being a burden for future partners saying,

*“But what does concern me is what you were saying earlier when we started is; I don’t have a partner and I’ve actually met a number of fellows and I sort of pushed them off, not because of different chemistry or things like that, I don’t want to be a burden to somebody else.” (Yellow, FG3)*.

Some participants described how they now needed to explain the effects of their prostate cancer treatment to potential sexual partners saying,

*“You know, I guess I’ve been honest with men that I’ve met up front. I tell them I do not get an erection, and especially when they’re within my age, they kind of get it, you know…maybe not cancer, but still you’re getting older” (Pink, FG1)*.

Some participants commented on how the effects of treatment had a profound impact on their approach to relationships, with one saying,

*“Again, you have to think out of the box a little bit and realize it’s an adjustment. There’s one good thing I read in a book, some guy was giving his testimonial and he said, “The erections aren’t the same, but what is the same? Life does change, life evolves, nothing stays the same.” That’s just the kind of philosophy I adapted, just thinking out of the box. And very good point; you’re getting pleasure, just a different way right now while you’re figuring it out with someone else.” (Pink, FG2)*.

Participants expressed gratitude and optimism for their life after treatment, with one saying,

*“I’m positive, my current PSA is kind of bounding, like the rest of them were talking about, I am going from like, .2 to .4, back to .2. Now, .02, undetectable, if it’s .02 to .04, so I’m very happy with that. It could be better, but I’m above ground, so I’m very, very grateful.” (Pink, FG1)*.

## Discussion

Our study addresses the increased calls for research into the unique experiences and considerations of MSM diagnosed with prostate cancer [11, 34]. MSM with prostate cancer experience bias in their healthcare encounters [13] have different concerns with prostate cancer treatment side effects than men who don’t identify as MSM [12, 35], and face unique social challenges including lack of support and challenges to their sexual identity. Our findings can be used to aid in the development of evidence-based interventions and counsel MSM more effectively after a prostate cancer diagnosis. One of the key experiences that the men in our focus groups discussed was their initial diagnosis. Prior studies have found that when receiving a diagnosis of prostate cancer, men experience a wide array of emotions including fear, shock, disbelief, and uncertainty [36]. One qualitative study of eight gay men with prostate cancer identified shock of diagnosis as a common theme [37]. The men in our groups had a wide range of reactions from hopelessness to shock, to an action-focused mindset which is consistent with prior studies. The wide range of reactions patients can experience when receiving a prostate cancer diagnosis underscores the importance approaching this conversation with intentional and empathic communication.

It was clear that the participants’ choice of provider and their interactions with the healthcare system were strongly impacted by their sexual identities. One participant felt so strongly about this that he described how one of the first things he disclosed to his physician is his sexual identity. This sentiment was not unique, as many men identified that they felt a gay physician was better able to understand their unique concerns, as well as relate to the different considerations they face. This finding is in line with prior studies which found that gay men experience difficulties disclosing their sexual identity to providers and encounter heteronormative assumptions [38]. Marginalized groups, including the LGBTQ + community experience significant stress in their lives. Minority stress theory provides one way of understanding how the biases these patients face in healthcare encounters leads to worse health outcomes. Power et al. surveyed LGBTQI people with cancer and concluded that minority stress compounds the impacts of other stressors associated with cancer [39]. It is critical to continue to study the barriers that MSM face during interactions with their healthcare providers and use that information to facilitate improved communication and clinical encounters in order to provide culturally competent care for MSM and improve health outcomes.

MSM have sexual concerns after prostate cancer treatment that are distinct from men who don’t identify as MSM. The participants in our study noted that they felt inadequate or that they had lost a part of themselves. Issues of erectile dysfunction, incontinence, and anejaculation led to changes in their sexual activity. Although sexual side effects themselves are an unfortunate part of prostate cancer treatment, several participants felt that there could have been improved anticipatory counseling on these side effects and thus they would have been more prepared. Prior studies have shown that MSM with prostate cancer have distinct sexual side effect concerns including the prostate as a source of pleasure, a significant role of ejaculate in the sexual experience, and a different erectile firmness required for anal sex compared to vaginal sex [21, 29, 40]. One survey study of gay men with prostate cancer by Hart et al. found a majority of participants reported substantial changes in sexual behavior after prostate cancer treatment, including a decrease in their role as the insertive partner [35]. Another mixed methods study of gay and bisexual men with prostate cancer found men had to make a significant change to their role in sex after treatment and found significant differences in quality of life outcomes based on role in sex leading them to conclude that shifting sexual behavior from insertive to receptive anal intercourse is associated with poorer sexual and mental health outcomes [41]. Our study is in agreement with these prior studies showing that MSM experience the sexual side effects of prostate cancer in a unique way. Mainwaring et al. reviewed 21 validated questionnaires relating to sexual dysfunction and its impact on quality of life and found that only one of them made mention of including MSM in the validation process [42]. It is critical that providers have the understanding and tools to address these differences so that they are better able to counsel MSM and their partners on treatment options and anticipatory guidance regarding side effects.

Sexual side effects can lead to significant social issues as well, with one study finding that some MSM had issues with identity, seeing themselves as less capable, and difficulty with social isolation or future relationships [25, 29]. Our findings support these studies with some of our participants noting that they felt a loss of identity, including one person noting a feeling of inadequacy, “feeling less of a man at this point”. Many of our unpartnered participants noted fear concerning future relationships. For MSM the social impact of prostate cancer can be profound. It is important for providers to understand the unique social consequences relating to prostate cancer diagnosis and treatment in MSM and address them when possible. We believe that improved pre-treatment counseling informed by our findings could better prepare MSM with prostate cancer for life after treatment.

This study is not without limitations. Notably, our cohort involved almost exclusive enrollment of white men. In our study, the focus groups were composed of 26 men, 25 of whom identified as white. Majority of our cohort earned a bachelor’s degree or higher level of education, potentially missing perspectives from patients with less formal education. Another potential limitation is not differentiating groups based on treatment type as there are different side effect profiles to consider for the different treatment modalities. We made the decision to ask on our intake forms, “to which gender are you most attracted sexually?” and “what is the gender of the people with whom you usually have sex?” rather than ask all participants to identify their gender and sexual identities. This is a weakness in that we do not know how our participants identify beyond MSM, but it is also a strength as the participants who chose to disclose their identities did so unprompted and we believe this approach allowed our participants to have a more active role in the flow of the discussion.

A strength of qualitative research is that no two groups are the same, as findings are influenced by the unique experiences and realities of the participants, moderators, and other environmental factors. Each focus group is impacted by the level of engagement of each participant, the number of participants, and the fact that different responses can promote different lines of discussion. Since qualitative research by definition does not set out to be inferential or generalizable, the findings here serve as an invaluable starting point for future lines of inquiry and lend further support to existing evidence of the distinct considerations for MSM with prostate cancer.

## Conclusion

In this study we found that MSM with prostate cancer have unique experiences compared to their non-MSM counterparts, including confronting the heterosexual biases of healthcare, concerns regarding post-treatment sexual function, and sexual side effects that can have profound impact on their self-esteem and relationships. It is clear that an important step toward reducing heteronormative bias in prostate cancer care is to better understand the goals, identity, and sexual practices of MSM and to provide informed anticipatory guidance. This study aids significantly in informing providers of the unique concerns of this patient population. The results of this work are consistent with prior studies and supports the growing magnitude of research identifying unique considerations of prostate cancer survivorship for MSM. We hope that future research can build off this study and the themes that were identified. This research is especially relevant given the number of MSM who are living with prostate cancer [16]. Future research areas include studies with intersectional perspectives and the development of targeted interventions to address the unique concerns of MSM with prostate cancer.

### Electronic supplementary material

Below is the link to the electronic supplementary material.

Additional file 1: Moderator’s guide. Focus group guide for moderators to use.

Additional file 2: Code Application. This file presents how unique codes were applied across each focus group and how the codes were grouped into higher-order categories and themes.

## Data Availability

Data sharing is not applicable to this article as no datasets were generated or analyzed during the current study.

## Abbreviations

MSM: Men who have sex with men
GBM: Gay or bisexual men
LGBQT+: Lesbian, gay, bisexual, transgender, queer or questioning, and more
US: United States
PCP: Primary care physician
GP: General practitioner
FG: Focus group
