# Supplementary material for: Heteronormative biases and distinctive experiences with prostate cancer among men who have sex with men: a qualitative focus group study
Source: BMC Urol. 2024 Jan 6;24:10. doi: 10.1186/s12894-023-01398-0 (PMC10771696; doi:10.1186/s12894-023-01398-0)
Supplement: Supplementary file 1 — Additional file 1: Moderator’s guide. Focus group guide for moderators to use. [file 12894_2023_1398_MOESM1_ESM.docx]

**SEXUAL FUNCTION IN MEN WHO HAVE SEX WITH MEN Focus Group Guide**

**Introductory Script for Focus Groups**

**Background**

Hello and welcome [*Introduce self and co-moderator*]

Thank you for taking the time to join our discussion about sexual function in men who have sex with men, with a focus on how prostate cancer may impact function. Everyone here today/tonight was invited because you have shown an interest in improving the health outcomes of men who have sex with men by improving the way sexual function outcomes are measured. Our goal is to work from that common ground and learn more from you about how to better understand sexual function in men who have sex with men, and how treatment for prostate cancer can impact sexual function.

We’re primarily interested in finding out about your experience, especially as it relates to how you think prostate cancer may or has affected your ability to function sexually, think clearly and remember things and aspects of mood, emotional and sexual health. There are no right or wrong answers, because everyone experiences things differently. Some of you may have had more negative experiences than others. We are interested in the full range of experiences, both positive and negative, so please feel free to share your point of view even if it differs from what others have said.

**Discussion Group Rules**

Before we begin, let me suggest some guidelines that will make our discussion more productive.

- - Please speak up—but only one person should talk at a time. We’re recording the session because we don’t want to miss any of your comments. If you have trouble hearing any of the comments, please let the group know.
  - In the discussion, please refer to one another by your number. In our reports of the results no names or identifying information will be attached to any comments. If your name is mentioned, it will be kept confidential.
  - Our role here is to ask questions and to listen. We’ll be summarizing information but won’t be actively participating in the conversation, only guiding it. We want you to feel free to talk to the group and not just to us. We’ll ask questions about the impact prostate cancer has or may have upon your quality of life. We are interested in your experiences, but because this is a research project, it is important that you link your comments back to the questions. We’ll move the discussion from one question to the next to try to keep us on track so that we can finish by [*insert time*].
  - We will present your compensation to you at the conclusion of the discussion.
  - Sometimes, people in focus groups think of things they want to say after the discussion has moved on to other questions. If you would like to add to your comments after the group, we will be around to talk with you privately.
  - Any questions before we begin?

# NOTE TO MODERATors: Focus Group Discussion Guide

This focus group discussion guide is presented in draft form. General guidelines are presented first followed by sample questions to stimulate discussion on the negative and positive effects of prostate cancer treatment on sexual function.

GENERAL

The interview discussion guide sets the agenda for the focus group discussion, but depending upon the flow of the discussions, the guide should flow as needed with the caveat that all necessary issues are covered by the end of the session. The discussion guide is generated from the research questions and goals of the focus groups.

When generating questions for the discussion guide, consider the following:

1. Start with more general questions first and move to more specific questions;
2. If sensitive topics are to be discussed (e.g. changes in sexual roles, etc), start with non-threatening, less personal issues and move to more personal and sensitive issues after the group has developed a rapport and trust among themselves and the moderator;
3. Questions should be presented in the order of importance, however this needs to be considered in light of points 1 and 2 and the length of time for the focus groups. (Focus group discussions should not extend past 2 hours – 90 minutes to 2 hours is optimal).
4. Questions should be open-ended, e.g. “How does {symptom} make you feel?” or “How does {symptom} affect you on a personally / socially / physically?”
5. Avoid introducing a response with the question (e.g. “Does having {PROBLEM IN DOMAIN AREA} make you angry?”) or providing leading questions (e.g. so you think that xxxx is a bad thing?”)
6. Don’t be afraid to follow-up on interesting issues that may not be included in the discussion guide. The point of qualitative research is as a theory-generating, fact-finding exercise, so if participants discuss issues that were not included in the discussion guide but are pertinent to the research questions and goals, follow their lead, but avoid getting lost on a tangential discussion that is not relevant to the research questions.
7. Keep the questions simple and meaningful; avoid long, complex sentences
8. Avoid potentially embarrassing or intimidating questions such as: “Why didn’t you follow your physician’s recommendations?” This could be asked as: “What factors interfered with you following your physician’s recommendations?”

In terms of the number of questions needed, this will vary depending upon the topic and patient group. If the group is to be widely heterogeneous, fewer questions may be needed as the group may offer great variation in responses. If the group is more homogeneous, the opposite may be true. Every focus group differs which is why discussion guides are guides which are meant to be flexible. The important thing for each focus group is to cover the essentials first.

**Questions:**

We would like to have everyone briefly introduce themselves, where you are from, whether you have a diagnosis of prostate cancer, how it was treated (if applicable) and how long it’s been since you completed treatment.

1. Assessment of global life changes:

**Tell us how your life has been affected by prostate cancer?**

Possible probes:

Probe: Please tell me what your life was like before prostate cancer.

Probe: How is your life different since your diagnosis and treatment?

Probe: Generally probe for details to get a complete narrative account of changes.

Probe: (if valence, not specified) Would you describe those changes as negative or positive?

Probe: (if all QOL domains not included) Did you experience any changes in the (physical, emotional, and sexual) aspect of your quality of life? If yes, can you tell me about those changes?

Probe: (if not described) Did you experience any positive changes?

Probe: What would you describe as the most significant long-lasting negative change in your life since your diagnosis and treatment?

Probe: What would you describe as the most significant long-lasting positive change in your life since your diagnosis and treatment?

1. Symptoms and side effects:

**Prostate cancer and its treatment can produce different sexual symptoms and side effects. What kinds were the most common for you?**

Possible probes:

Probe: Make sure to probe sexual, bowel, bladder dysfunction

Probe: How did [insert side effect] affect your quality of life?

Probe: What side effects were the most burdensome for you?

Probe: What side effects went away vs. ones that continue to bother you?

1. Ways of Coping

**How did you (or would you) deal with or manage with changes in sexual function?**

Possible probes:

Probe: What kind of information did you receive from your medical provider about changes in sexual function?

Probe: How did the medical team help prepare you for this side effect?

Probe: Who did you talk with to learn how to manage this side effect?

Probe: What would you suggest to another gay or bisexual man with sexual dysfunction in terms of how to deal with it?

1. Thoughts on currently existing sexual function questionnaires:

**Please take a moment to review these questionnaires that currently assess sexual function in men after treatment for prostate cancer.**

[Provide patients with EPIC Sexual function questionnaire, PROMIS sexual function measures, MSHQ]

Possible probes:

Probe: Which questions on these questionnaires apply most to you?

Probe: When thinking about sexual function domains relevant to men who have sex with men, can you think of questions or domains regarding sexual function that are not included in these questionnaires?

Probe: If prostate stimulation or sensitivity is decreased after treatment for prostate cancer, how important do you think it would be to measure this domain?

Probe: What areas relating to prostate function and receptive anal intercourse do you think would be important to touch upon?

1. Final thoughts/questions

**Is there anything else that you think is important for me to know about your experience that I did not ask you about?**

**Thank you for your time.**
